# Diagnostic Trends of Minors in Psychiatric Emergency Care: An Observational Study

**DOI:** 10.62641/aep.v54i1.2061

**Published:** 2026-02-15

**Authors:** Andrea Jiménez-Mayoral, Dídac Florensa, Vicent Llorca-Bofí, María Irigoyen-Otiñano

**Affiliations:** ^1^Psychiatry Service, Santa Maria University Hospital, 25198 Lleida, Spain; ^2^Group of Biological Functionings of Mental Disorders, Institute for Biomedical Research in Lleida (IRB), 25198 Lleida, Spain; ^3^Department of Computer Engineering, University of Lleida, 25001 Lleida, Spain; ^4^Barcelona Clinic Schizophrenia Unit (BCSU), Institute of Biomedical Research August Pi i Sunyer (IDIBAPS), Biomedical Research Networking Center for Mental Health, Carlos III Health Institute (CIBERSAM), 08036 Barcelona, Spain; ^5^Department of Medicine, University of Barcelona, 08036 Barcelona, Spain; ^6^CIBERSAM Group 10, Center for Biomedical Research in Mental Health Network (CIBERSAM), 28029 Madrid, Spain

**Keywords:** adolescent, child, emergency services, psychiatric, mental health, diagnosis

## Abstract

**Background:**

Diagnostic stability in child and adolescent psychiatry is a key indicator of validity and essential for clinical decision-making. Few longitudinal studies have examined diagnostic trajectories after a first emergency psychiatric contact.

**Methods:**

We conducted a retrospective observational cohort study at Santa Maria University Hospital (Lleida, Spain). A total of 583 patients aged 4–18 years presenting for their first psychiatric emergency visit between 2017 and 2023 were included, with 24-month follow-up. Sociodemographic and clinical data were extracted from Electronic Health Records. Diagnostic transitions were summarized using transition matrices. An exploratory association analysis (Apriori algorithm) identified frequent T1→T2 patterns, reported with confidence and lift. Diagnostic stability was defined as the proportion of patients retaining the same diagnosis at follow-up.

**Results:**

Median age at baseline 14.9 years (interquartile range [13–16]); 54.55% were female. Schizophrenia/psychosis showed the highest stability (71%), followed by intellectual disability with gender identity disorder (67%). Mood disorders showed moderate stability (~44%), while others such as eating disorders (26%) or conduct disorders (17%) had lower stability. The strongest associations were “no prior diagnosis → eating disorder” (confidence = 1.00; lift = 12.76) and “autism spectrum disorder + attention-deficit/hyperactivity disorder (ADHD) → conduct disorders” (confidence = 0.66; lift = 2.55).

**Conclusions:**

Diagnostic stability is heterogeneous, with high persistence in schizophrenia/psychosis and low in eating disorders and ADHD. Association analysis identified specific trajectories that may help anticipate clinical evolution. Findings highlight the importance of longitudinal evaluation in early psychiatric care.

## Introduction

Mental disorders affect around 15% of children and adolescents and have a long-term impact on adult life [1, 2, 3]. Numerous studies have examined the consequences of early psychiatric diagnoses, showing that more than half of adults with mental disorders had received a diagnosis before the age of 15, and nearly 80% by young adulthood [4, 5, 6, 7].

Over the last decade, almost 50% of minors have entered the mental health care system through emergency departments, which serve as a primary gateway to specialized psychiatric care for children and adolescents [8, 9, 10, 11]. In addition to the challenges inherent to first-contact situations, emergency psychiatric care presents further difficulties: limited evaluation time, insufficient specific training and experience among emergency psychiatrists, lack of standardized diagnostic tools, and service overload [12, 13, 14, 15].

Consequently, the accuracy and thoroughness of diagnostic assessments conducted in emergency settings have gained increasing importance, especially as demand for emergency mental health services for minors has grown substantially in recent years [16, 17, 18]. This rise has been accompanied by notable changes in diagnostic profiles and in the stability of initial diagnoses in emergency care units [19, 20, 21]. Currently, the most frequent presenting problems include behavioural disorders, affective disorders (such as depression and bipolar disorder), and self-injurious behaviours [22].

Mental disorders in children and adolescents can follow diverse developmental trajectories, such as remission, changes in diagnosis, or the emergence of multiple comorbid conditions, often displaying a heterotypic pattern [19, 20, 23]. Clinical evaluation, semi-structured interviews, and follow-up assessments after the emergency visit—together with the identification of potential diagnostic co-occurrence—play a decisive role in shaping the clinical course and diagnostic stability [19, 23, 24]. In fact, diagnostic changes occur more frequently following emergency evaluations than after outpatient consultations, as minors are often assessed during periods of greater vulnerability and psychopathological decompensation [20, 21, 22].

This study aimed to describe the evolution and stability of psychiatric diagnoses over time among minors presenting for their first psychiatric emergency assessment.

## Materials and Methods

### Study Design and Setting

We conducted an observational, longitudinal cohort study at the Department of Psychiatry, Santa Maria University Hospital (Lleida, Spain). The study period ranged from June 2017 to June 2023, with a median of 24-month follow-up. The primary objective was to evaluate diagnostic stability and changes in psychiatric disorders among children and adolescents after their first emergency contact.

### Data Sources and Measurement

The study relies on retrospective extraction of longitudinal information, as all data were generated prior to the initiation of the study. All clinical and sociodemographic information was obtained from the electronic health records (EHR) of the Emergency Department of Psychiatry. Data were fully anonymized before analysis, and information was retrieved through a manual review of each individual episode. All collection included baseline, intermediate, and follow-up diagnoses, as well as details of emergency visits and admissions when available. This single-center design allowed for uniform diagnostic assessment and consistent data capture.

### Participants

Eligible participants were children and adolescents aged 4–18 years who had their first contact with the Emergency Department of Psychiatry through the psychiatric emergency room during the study period. All patients were residents within the hospital’s catchment area and covered by the national public health system; therefore, race/ethnicity and payment method are not routinely recorded in our clinical EHR system. Exclusion criteria were loss to follow-up due to emigration or death.

Eligible individuals were: (1) Children and adolescents aged 4–18 years who presented their first psychiatric emergency visit during the study period. (2) Those with at least one documented follow-up evaluation within 24 months after the index visit. (3) Those presenting with psychiatric symptoms warranting emergency consultation (e.g., mood, behavioural, psychotic, anxiety, or neurodevelopmental symptoms).

Exclusion criteria were: (1) Diagnosed or suspected severe neurological or systemic medical conditions explaining psychiatric symptoms (e.g., epilepsy, autoimmune encephalitis). (2) Somatic diseases with primary medical rather than psychiatric management. (3) Duplicate or repeated visits of the same patient, with only the first contact was retained. (4) Missing or unverified diagnostic information, or loss to follow-up due to emigration or death.

### Variables

The exposure was defined as the primary psychiatric diagnosis at baseline (T1), corresponding to the first psychiatric emergency department visit. The outcome was the primary diagnosis at follow-up (T2), established 24 months later. Intermediate diagnoses during subsequent visits were also recorded to evaluate diagnostic trajectories. Psychiatric diagnoses were coded according to the *Diagnostic and Statistical Manual of Mental Disorders, Fourth Edition* (DSM-IV), as required for billing and classification within the EHR system. All diagnoses were cross-checked and confirmed independently by senior psychiatrists of the Department of Psychiatry to ensure diagnostic accuracy. Cases coded as “diagnosis deferred” corresponded to situations in which the clinical evaluation was inconclusive or insufficient to assign a specific diagnostic category during the emergency visit. For the descriptive data associated with T1, the following sociodemographic variable was included: sex, geographic origin, household and previous mental health follow-up. These variables were used exclusively to describe the profile of the patients included at the T1 assessment point.

### Statistical Analysis

All analyses were descriptive in nature and focused on diagnostic stability and change over time. First, diagnostic transition matrices were constructed to quantify co-occurrences between baseline (T1) and follow-up (T2) diagnoses; these were visualized using heatmaps. Second, we conducted an exploratory association analysis of T1→T2 transitions, implemented using the Apriori algorithm. Results were summarized with confidence (the conditional probability of T2 given T1) and lift (the relative strength of association compared with chance). The Apriori algorithm was implemented using the mlxtend Python package, with minimum support and confidence thresholds set at 0.05 and 0.6, respectively, following common conventions for clinical association mining. By convention, a lift value of 1.0 indicates independence, values above 1.0 indicate positive association, and values greater than 2.0 are typically interpreted as strong. Third, diagnostic stability was quantified as the proportion of patients retaining the same diagnosis (or diagnostic family) at follow-up. For interpretative purposes, stability values >70% were considered high, 40%–70% moderate, and <40% low, consistent with prior literature on diagnostic reliability. All analyses were performed using Python. Missing data were minimal (<5%) and restricted to patients lost to follow-up, who were excluded by design. No data imputation was performed.

## Results

We included 583 children and adolescents who presented for their first psychiatric emergency visit between June 2017 and June 2023. The median age at index visit was 14.9 years (interquartile range [13–16]), and 54.55% were female (male [45,45] %). The distribution of baseline diagnostic categories is summarized in Table 1. Diagnostic transitions, exploratory association patterns, and diagnostic stability over the 24-month follow-up.

**Table 1.** **Baseline characteristics of the study sample**.

Characteristics		Total (N = 583)
Age at first emergency visit, years		Median (IQR) = 14.9 (13–16)
Sex, n (%)		
	Female	318 (54.55%)
	Male	265 (45.45%)
Geographical origin, n (%)		
	Spanish	413 (70.84%)
	Other European	31 (5.32%)
	American	43 (7.37%)
	African	78 (13.38%)
	Asian	9 (1.54%)
	Not registered	9 (1.54%)
Household, n (%)		
	Family of origin	326 (55.92%)
	Extended family	130 (22.30%)
	Residential care centers for minors	119 (20.41%)
	Foster family	5 (0.86%)
	Not registered	3 (0.51%)
Previous mental health follow-up, n (%)		
	Yes	159 (27.27%)
	No	424 (72.73%)
Year of first visit, n (%)		
	2017	92 (15.78%)
	2018	154 (26.41%)
	2019	149 (25.56%)
	2020	86 (14.75%)
	2021	102 (17.50%)
Primary diagnosis at baseline (T1), n (%)		
	SCZ	7 (1.20%)
	ND including ASD, ADHD and intellectual disability	95 (16.29%)
	MD	85 (14.58%)
	ED	46 (7.89%)
	SUD	9 (1.54%)
	CD	89 (15.27%)
	AD	90 (15.45%)
	PD	9 (1.54%)
	Sleep disorders	2 (0.34%)
	GID	1 (0.17%)
	DD	150 (25.73%)

Note: Sociodemographic and clinical characteristics of the 583 children and adolescents at first psychiatric emergency contact. Values are expressed as n (%) unless otherwise indicated. Abbreviations: IQR, interquartile range. Abbreviations: IQR, interquartile range; SCZ, Schizophrenia and psychosis disorders; ND, Neurodevelopmental disorders; ASD, autism spectrum disorder; ADHD, attention deficit/hyperactivity disorder; MD, Mood disorders; ED, Eating disorders; SUD, Substance use disorders; CD, Conduct disorders; AD, Anxiety and Neurotic Disorders; PD, Personality disorders; GID, Gender Identity Disorder; DD, Diagnosis Deferred.

### Diagnostic Transitions

**Fig. 1.**
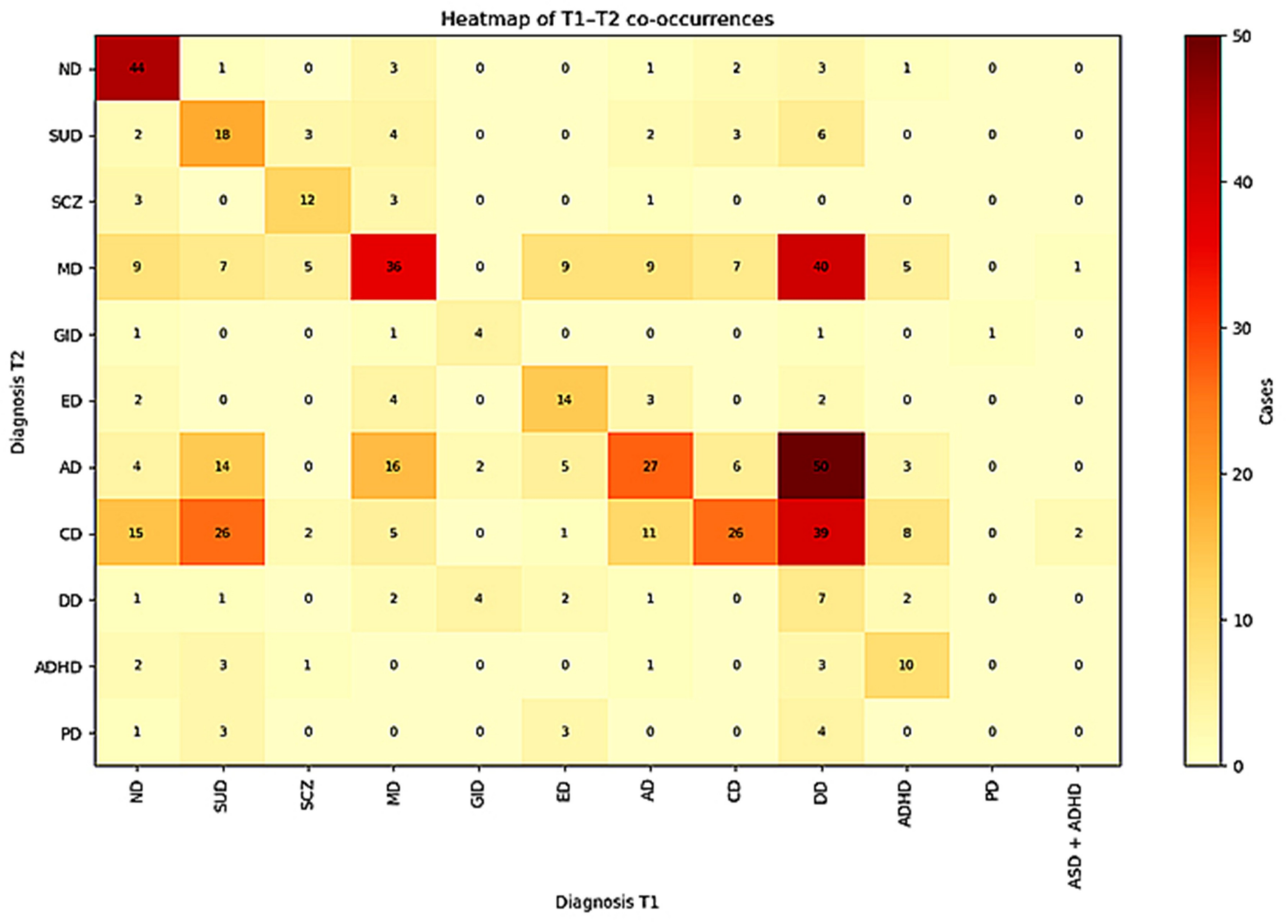
**Transition matrix of baseline (T1) and follow-up (T2) diagnoses**. The heatmap represents the distribution of diagnostic categories between baseline (T1) and follow-up (T2). Darker cells along the diagonal indicate higher diagnostic stability. ND, Neurodevelopmental Disorder; SUD, substance use disorder; SCZ, Schizophrenia and Psychosis; MD, Mood Disorder; GID, gender identity disorder; ED, eating disorder; AD, Anxiety and other Neurotic Disorders; CD, Conduct Disorder; DD, Diagnosis Deferred; ADHD, Attention-Deficit/Hyperactivity Disorder; PD, Personality Disorder; ASD, autism spectrum disorder.

The transition matrix (visualized as a heatmap) revealed heterogeneous diagnostic continuity across categories. The largest diagonal cells—indicating higher stability—corresponded to neurodevelopmental disorders (*n* = 44), mood disorders (*n* = 36), anxiety disorders (*n* = 27), and conduct disorders (*n* = 26). Several categories (e.g., mood disorders, anxiety disorders, conduct disorders) frequently transitioned to diagnosis deferred at T2. The transition matrix is summarized in Fig. 1.

### Association Rule Mining

The exploratory association analysis (Apriori algorithm) identified a small set of frequent T1→T2 transitions. The strongest rule by lift was “no prior diagnosis → eating disorder” (confidence = 1.00; lift = 12.76). “Autism spectrum disorder (ASD) combined with attention-deficit/hyperactivity disorder (ADHD) → conduct disorders” also showed a strong association (confidence = 0.66; lift = 2.55). Additional rules had modest lifts (≈1.1–1.2), including “Personality Disorders → Diagnosis Deferred” (confidence = 0.33; lift = 1.16), “ASD + ADHD → Mood Disorders” (confidence = 0.33; lift = 1.17) and “SUD → Conduct Disorders” (confidence = 0.31; lift = 1.19). The evolution of diagnostic transitions is summarized in Table 2.

**Table 2.** **Exploratory association analysis of diagnostic transitions**.

Prior diagnosis	Subsequent diagnosis	Confidence	Lift
SUD	CD	0.31	1.19
ASD + ADHD	MD	0.33	1.17
GID	DD	0.35	1.24
None/Not Recorded	ED	1.0	12.76
AD	DD	0.32	1.14
ASD + ADHD	CD	0.66	2.55
PD	DD	0.33	1.16

Note: Results of rule-based association analysis (Apriori algorithm) between baseline (T1) and follow-up (T2) diagnoses. Confidence expresses the conditional probability of the consequent diagnosis given the antecedent. Lift indicates the strength of association compared with independence (lift = 1.0). Values >1.0 indicate positive association; values >2.0 are considered strong. Abbreviations: ASD, autism spectrum disorder; ADHD, attentiondeficit/ hyperactivity disorder; SUD, substance use disorder; CD, Conduct Disorders; MD, Mood Disorders; GID, Gender Identity Disorder; DD, Diagnosis Deferred; ED, Eating Disorders; AD, Anxiety and Neurotic Disorders; PD, Personality Disorders.

### Diagnostic Stability

Stability percentages ranged from 0% to 71%. Schizophrenia/psychosis demonstrated high stability (71%). Mood disorders showed moderate stability (~44%), while eating disorders displayed low stability (anorexia nervosa ~26%; bulimia nervosa ~20%). The category “None/Not recorded” showed very low stability (4%). Some trajectories, such as dysfunctional personality traits with somatoform disorders, did not maintain diagnostic continuity (0%). Stability of diagnostic categories is summarized in Table 3.

**Table 3.** **Diagnostic stability by baseline category**.

Prior diagnosis	Emergency primary diagnosis	Current primary diagnosis	Stability percentage
SCZ	SUD	SCZ	0.71
Intellectual disability	GID	ED (Anorexia nervosa)	0.67
GID	SUD	MD	0.44
SUD	SCZ	MD	0.44
ED (anorexia nervosa)	Sleep disorders	ED (Bulimia nervosa)	0.26
MD	Sleep disorders	CD	0.21
ASD and ADHD	ED (Bulimia nervosa)	Eating Disorder (Bulimia nervosa)	0.20
CD	ED (Anorexia nervosa)	CD	0.17
Sleep disorders	ED (Bulimia nervosa)	CD	0.12
None/Not Recorded	Sleep disorders	CD	0.04
PD	Somatoform disorders	GID	0.00

Note: Proportion of patients who retained the same diagnosis (or diagnostic family) at follow-up (T2). Stability percentages correspond to diagonal probabilities in the transition matrix. By convention, stability was categorized as high (>70%), moderate (40–70%), or low (<40%). ASD, autism spectrum disorder; ADHD, attention-deficit/hyperactivity disorder; SUD, substance use disorder; CD, conduct disorder. Abbreviations: ASD, autism spectrum disorder; ADHD, attention-deficit/hyperactivity disorder; SUD, substance use disorder; CD, conduct disorder; SCZ, Schizophrenia and Psychosis; GID, Gender Identity Disorder; ED, Eating disorder; MD, Mood disorder; PD, Personality Disorder.

## Discussion

The results of this study provide important insights into the diagnostic stability and patterns of comorbidity among mental health disorders in minors treated at a provincial emergency care hospital. Our findings reveal substantial variability in the continuity of diagnoses across categories, with certain disorders exhibiting high diagnostic stability, while others demonstrate marked diagnostic shifts or associations with the absence of a psychiatric condition at follow-up.

One of the most robust findings is the high diagnostic stability observed in neurodevelopmental disorders. This finding aligns with prior research indicating that neurodevelopmental disorders typically follow stable, long-term courses and therefore require sustained management and support [7, 20, 21]. The strong association between neurodevelopmental disorders and conduct disorders further suggests that neurodevelopmental vulnerabilities, particularly when compounded by attentional or behavioural dysregulation, may predispose individuals to impaired impulse control [25, 26, 27]. The associations observed between the two previous disorders with substance use disorders suggest a complex interplay of genetic, developmental, and environmental influences that shape diagnostic trajectories. In addition, the moderate link between substance use disorder and impulse control disorder supports existing evidence of co-occurrence between substance use and impulse control difficulties during adolescence [7, 27, 28]. A similar situation regarding diagnostic stability was observed in the case of diagnoses of schizophrenia and psychosis. These conditions exhibited strong continuity, with a substantial proportion of patients retaining their initial diagnosis at follow-up. These findings are consistent with strong studies on diagnostic stability within the child and adolescent psychosis spectrum [28, 29, 30]. Also notable is the marked stability of substance use disorders, and its strong relationship with co-occurrence of schizophrenia and psychosis at the end of follow-up. This reflects the chronic and relapsing trajectory that often characterizes these conditions and highlights the need for ongoing monitoring and interventions specifically designed for young people [30, 31].

In contrast, both of affective disorders and neurosis, displayed a mixed pattern of diagnostic stability, with some cases evolving toward the absence of psychopathology at the end of follow-up in this work. This outcome may be explained by natural recovery processes, the efficacy of acute interventions, or fluctuations in symptom severity [32, 33]. Additionally, affective disorders—particularly in childhood and adolescence—may evolve into or coexist with other psychopathologies, a phenomenon reflected in the observed diagnostic overlap between affective disorders and impulse conduct disorders [34]. In addition, the category coded as diagnosis deferred showed very low stability, reflecting the clinical reality that emergency assessments often take place under conditions of uncertainty. This finding reinforces the importance of maintaining diagnostic flexibility and ensuring longitudinal reassessment to capture the evolving nature of psychopathology in minors.

Gender identity disorder exhibited particularly low diagnostic stability; a finding likely attributable to the developmental nature of gender identity during adolescence. As adolescence represents a critical period of gender identity exploration, diagnostic changes may reflect this normative developmental process rather than persistent psychopathology [35]. Notably, gender identity disorderwas also uniquely associated with the subsequent absence of psychiatric diagnoses, suggesting that for some minors, gender-related distress may resolve over time without evolving into enduring psychopathology. This observation underscores the need for further longitudinal studies on gender identity development and its mental health correlates in youth populations [36].

Low diagnostic stability was observed in eating disorders (particularly anorexia nervosa), conditions prone to symptom fluctuations and diagnostic reclassification. Eating disorders, in particular, frequently coexist with other psychiatric illnesses and can evolve in response to psychological or social stressors [8, 37]. However, it should be noted that this study found that a large proportion of patients ultimately diagnosed with eating disorders, at baseline and during follow-up, did not have a confirmed primary diagnosis. This could be explained by the difficulty of diagnosis in the early stages of the disease, the rapid onset of these disorders, or the lack of psychopathological exploration of the eating sphere during evaluation in a mental health emergency department. Early identification and multidimensional assessment are essential to improve diagnostic precision in conditions with low stability in minors. Integrating biological, psychological, and social factors while considering developmental trajectories helps distinguish transient from persistent cases, preventing overdiagnosis and inappropriate interventions. Anyway, limited evidence underscores the need for longitudinal, rigorous research to inform ethically sound and developmentally appropriate clinical practice [11, 12, 13]. This study needs to be expanded to determine whether there are risk factors related to the late diagnosis of these disorders.

Several limitations should be considered. While some disorders were associated with the absence of psychopathology at follow-up (mood disorders, neurosis, conduct disorders), this outcome must be interpreted with caution. Such findings may not reflect true symptomatic remission but rather the effects of diagnostic reclassification, symptom variability, or loss to follow-up. The lack of a diagnosis of eating disorders at baseline and during follow-up could be influenced by the conditions under which clinical interviews are conducted in an emergency department, which require speed and do not usually focus on eating-related symptoms unless they are the primary reason for the consultation. Also, this single-center design allowed for uniform diagnostic assessment and consistent data capture although it may limit the generalizability of findings to other clinical settings.

## Conclusions

This study revealed substantial variability in diagnostic stability among minors in psychiatric emergency care. Neurodevelopmental, psychotic, and substance use disorders showed higher stability, whereas affective, neurotic, gender identity disorder, and eating disorders were more prone to diagnostic change. These findings emphasize the need for longitudinal, developmentally informed assessments and consistent follow-up to improve diagnostic reliability and clinical outcomes. Maintaining flexibility during emergency evaluations and incorporating periodic reassessment into clinical protocols may better capture the evolving nature of psychopathology in youth. Further research is warranted to clarify the longitudinal course and determinants of diagnostic instability, particularly in eating disorders.

## Availability of Data and Materials

We conducted an observational cohort study at Santa Maria University Hospital (Lleida, Spain). A total of 583 patients aged 4–18 years presenting for their first psychiatric emergency visit between 2017 and 2023 were included, with 24-month follow-up. Sociodemographic and clinical data were extracted from electronic health records. The datasets generated and/or analyzed during the current study are not publicly available because they contain confidential patient information and are subject to ethical and privacy restrictions, but are available from the corresponding author on reasonable request.

## References

[1] Göbel K, Ortelbach N, Cohrdes C, Baumgarten F, Meyrose AK, Ravens-Sieberer U (2022). Co-occurrence, stability and manifestation of child and adolescent mental health problems: a latent transition analysis. *BMC Psychology*.

[2] Dalsgaard S, Thorsteinsson E, Trabjerg BB, Schullehner J, Plana-Ripoll O, Brikell I (2020). Incidence Rates and Cumulative Incidences of the Full Spectrum of Diagnosed Mental Disorders in Childhood and Adolescence. *JAMA Psychiatry*.

[3] Sacco R, Camilleri N, Eberhardt J, Umla-Runge K, Newbury-Birch D (2024). A systematic review and meta-analysis on the prevalence of mental disorders among children and adolescents in Europe. *European Child & Adolescent Psychiatry*.

[4] Copeland W, Shanahan L, Costello EJ, Angold A (2011). Cumulative Prevalence of Psychiatric Disorders by Young Adulthood: a Prospective Cohort Analysis from the Great Smoky Mountains Study. *Journal of the American Academy of Child and Adolescent Psychiatry*.

[5] Whitbeck LB, Yu M, Johnson KD, Hoyt DR, Walls ML (2008). Diagnostic Prevalence Rates from Early to Mid-Adolescence among Indigenous Adolescents: first Results from a Longitudinal Study. *Journal of the American Academy of Child and Adolescent Psychiatry*.

[6] Chan SSM, Wong OWH, Hussain S, Tsoi KKF, Ma KKY, Chau SWH (2025). Twelve-month prevalence of DSM-5 mental disorders and the psychosocial correlates- a child and adolescent psychiatric epidemiologic survey in Hong Kong SAR. The Lancet regional health. *Western Pacific*.

[7] Solmi M, Radua J, Olivola M, Croce E, Soardo L, Salazar de Pablo G (2022). Age at onset of mental disorders worldwide: large-scale meta-analysis of 192 epidemiological studies. *Molecular Psychiatry*.

[8] Kalb LG, Stapp EK, Ballard ED, Holingue C, Keefer A, Riley A (2019). Trends in Psychiatric Emergency Department Visits among Youth and Young Adults in the us. *Pediatrics*.

[9] Bommersbach TJ, McKean AJ, Olfson M, Rhee TG (2023). National Trends in Mental Health-Related Emergency Department Visits among Youth, 2011-2020. *JAMA*.

[10] Chun TH, Duffy SJ, Grupp-Phelan J (2019). The Increasing Burden of Psychiatric Emergencies: a Call to Action. *Pediatrics*.

[11] Gill PJ, Saunders N, Gandhi S, Gonzalez A, Kurdyak P, Vigod S (2017). Emergency Department as a first Contact for Mental Health Problems in Children and Youth. *Journal of the American Academy of Child and Adolescent Psychiatry*.

[12] So P, Nooteboom LA, Vullings RM, Mulder CL, Vermeiren R (2024). Psychiatric emergency consultations of minors: a qualitative study of professionals’ experiences. *BMC Psychiatry*.

[13] Hendriks G, Tan C, Vicknesan MJ, Chen HY, Sung SC, Ang ASY (2024). Physician perceptions of medically unexplained symptoms in adolescent patients presenting to the emergency department. *Asian Journal of Psychiatry*.

[14] Newton AS, Soleimani A, Kirkland SW, Gokiert RJ (2017). A Systematic Review of Instruments to Identify Mental Health and Substance Use Problems among Children in the Emergency Department. *Academic Emergency Medicine*.

[15] Saidinejad M, Duffy S, Wallin D, Hoffmann JA, Joseph M, Uhlenbrock JS (2023). The Management of Children and Youth with Pediatric Mental and Behavioral Health Emergencies. *Journal of Emergency Nursing*.

[16] Riesgo Rubio A, Seijo Zazo E (2024). Urgencias psiquiátricas en población infanto-juvenil. *Boletín de Pediatría*.

[17] Dror C, Hertz-Palmor N, Yadan-Barzilai Y, Saker T, Kritchmann-Lupo M, Bloch Y (2022). Increase in Referrals of Children and Adolescents to the Psychiatric Emergency Room Is Evident Only in the Second Year of the COVID-19 Pandemic-Evaluating 9156 Visits from 2010 through 2021 in a Single Psychiatric Emergency Room. *International Journal of Environmental Research and Public Health*.

[18] Adrados-Pérez M, Llorca-Bofí V, Mur-Laín M, Albert-Porcar C, Nicolau-Subires E, Ibarra-Pertusa L (2023). Trajectories of children and adolescents attending a psychiatric emergency unit during the COVID-19 confinements: 2020-2022 longitudinal study. *Child and Adolescent Psychiatry and Mental Health*.

[19] Krantz MF, Dalsgaard S, Osler M, Jorgensen MB, Jorgensen A, Jørgensen TSH (2025). Diagnostic trajectories and stability of mental disorders in childhood and adolescence - a nation-wide cohort study using sequence analysis. *European Psychiatry*.

[20] Boyer J, Cautenet A, Ligier F (2021). The evolution of child psychiatry emergencies: Results and reflections from a Nancy University Hospital study. *Encephale*.

[21] Hagmann D, Allgaier K, Wolf J, Chiumento O, Bürkle L, Conzelmann A (2022). Evolution of Emergency Department Characteristics in Child and Adolescent Psychiatry: A Retrospective Review over Two Decades. *Zeitschrift fur Kinder-und Jugendpsychiatrie und Psychotherapie*.

[22] Poyraz Fındık OT, Fadıloğlu E, Ay P, Fiş NP (2022). Emergency mental health care for chi̇ldren and adolescents outside of regular working hours: 7 years outcomes from a tertiary hospital. *Asian Journal of Psychiatry*.

[23] Girela-Serrano B, Miguélez-Fernández C, Abascal-Peiró S, Peñuelas-Calvo I, Jiménez-Muñoz L, Moreno M (2024). Diagnostic trajectories of mental disorders in children and adolescents: a cohort study. *European Child & Adolescent Psychiatry*.

[24] Iannuzzi D, Hall M, Oreskovic NM, Aryee E, Broder-Fingert S, Perrin JM (2022). Emergency Department Utilization of Adolescents and Young Adults with Autism Spectrum Disorder. *Journal of Autism and Developmental Disorders*.

[25] Neuhaus E, Osuna A, Tagavi DM, Shah-Hosseini S, Simmons S, Gerdts J (2022). Clinical Characteristics of Youth with Autism or Developmental Disability during Inpatient Psychiatric Admission. *Journal of Clinical Medicine*.

[26] Estric C, Calati R, Lopez-Castroman J (2022). Adverse Childhood Experiences and Neurocognition in Borderline Personality Disorder: a Call-to-Action Perspective Review. *Harvard Review of Psychiatry*.

[27] Hollis C (2000). Adult Outcomes of Child- and Adolescent-Onset Schizophrenia: Diagnostic Stability and Predictive Validity. *The American Journal of Psychiatry*.

[28] Correll CU, Arango C, Fagerlund B, Galderisi S, Kas MJ, Leucht S (2024). Identification and treatment of individuals with childhood-onset and early-onset schizophrenia. *European Neuropsychopharmacology*.

[29] Patel PK, Leathem LD, Currin DL, Karlsgodt KH (2021). Adolescent Neurodevelopment and Vulnerability to Psychosis. *Biological Psychiatry*.

[30] Pelizza L, Leuci E, Leucci AC, Quattrone E, Azzali S, Pupo S (2024). Diagnostic shift in first episode psychosis: Results from the 2-year follow-up of the “Parma Early Psychosis” program. *Schizophrenia Research*.

[31] Edbrooke-Childs J, Wolpert M, Zamperoni V, Napoleone E, Bear H (2018). Evaluation of reliable improvement rates in depression and anxiety at the end of treatment in adolescents. *BJPsych Open*.

[32] Bear HA, Edbrooke-Childs J, Norton S, Krause KR, Wolpert M (2020). Systematic Review and Meta-analysis: Outcomes of Routine Specialist Mental Health Care for Young People with Depression and/or Anxiety. *Journal of the American Academy of Child and Adolescent Psychiatry*.

[33] Wolff JC, Ollendick TH (2006). The Comorbidity of Conduct Problems and Depression in Childhood and Adolescence. *Clinical Child and Family Psychology Review*.

[34] Park RJ, Goodyer IM (2000). Clinical guidelines for depressive disorders in childhood and adolescence. *European Child & Adolescent Psychiatry*.

[35] Kaltiala-Heino R, Sumia M, Työläjärvi M, Lindberg N (2015). Two years of gender identity service for minors: overrepresentation of natal girls with severe problems in adolescent development. *Child and Adolescent Psychiatry and Mental Health*.

[36] Morandini JS, Kelly A, de Graaf NM, Malouf P, Guerin E, Dar-Nimrod I (2023). Is Social Gender Transition Associated with Mental Health Status in Children and Adolescents with Gender Dysphoria?. *Archives of Sexual Behavior*.

[37] Lin BY, Liu A, Xie H, Eddington S, Moog D, Xu KY (2024). Co-occurring psychiatric disorders in young people with eating disorders: an multi-state and real-time analysis of real-world administrative data. *General Hospital Psychiatry*.

